# Assessment of the kidney function parameters split function, mean transit time, and outflow efficiency using dynamic FDG-PET/MRI in healthy subjects

**DOI:** 10.1186/s41824-019-0051-9

**Published:** 2019-02-15

**Authors:** Barbara K. Geist, Pascal Baltzer, Barbara Fueger, Martina Hamboeck, Thomas Nakuz, Laszlo Papp, Sazan Rasul, Lalith Kumar Shiyam Sundar, Marcus Hacker, Anton Staudenherz

**Affiliations:** 1 Department of Biomedical Imaging and Image-guided Therapy, Division of Nuclear Medicine, Medical University of Vienna, Waehringer Guertel 18-20, 1090 Vienna, Austria; 2 Department of Biomedical Imaging and Image-guided Therapy, Division of General and Pediatric Radiology, Medical University of Vienna, Vienna, Austria; 3 Center for Medical Physics and Biomedical Engineering, Medical University of Vienna, Vienna, Austria

**Keywords:** FDG, PET/MRI, Renal split function, Renal mean transit time, Renal outflow efficiency

## Abstract

**Background:**

Traditionally, isotope nephrography is considered as the method of choice to assess kidney function parameters in nuclear medicine. We propose a novel approach to determine the split function (SF), mean transit time (MTT), and outflow efficiency (OE) with 2-deoxy-2-[18F]fluoro-D-glucose (FDG) dynamic positron emission tomography (PET).

**Materials and methods:**

Healthy adult subjects underwent dynamic simultaneous FDG-PET and magnetic resonance imaging (PET/MRI). Time-activity curves (TACs) of total kidneys, renal cortices, and the aorta were prospectively obtained from dynamic PET series. MRI images were used for anatomical correlation. The same individuals were subjected to dynamic renal Technetium-99 m-mercaptoacetyltriglycine (MAG3) scintigraphy and TACs of kidneys; the perirenal background and the left ventricle were determined. SF was calculated on the basis of integrals over the TACs, MTT was determined from renal retention functions after deconvolution analysis, and OE was determined from MTT. Values obtained from PET series were compared with scintigraphic parameters, which served as the reference.

**Results:**

Twenty-four subjects underwent both examinations. Total kidney SF, MTT, and OE as estimated by dynamic PET/MRI correlated to their reference values by *r* = 0.75, *r* = 0.74 and *r* = 0.81, respectively, with significant difference (*p* < 0.0001) between the means of MTT and OE. No correlations were found for cortex FDG values.

**Conclusions:**

The study proofs the concept that SF, MTT, and OE can be estimated with dynamic FDG PET/MRI scans in healthy kidneys. This has advantages for patients receiving a routine PET/MRI scan, as kidney parameters can be estimated simultaneously to functional and morphological imaging with high accuracy.

## Background

Renal split function (SF), mean transit time (MTT), and outflow efficiency (OE) obtained from renal scintigraphy are kidney function parameters with high clinical relevance for the detection of renal pathologies including obstruction or renovascular hypertension, assessment of potential live donors for kidney transplantation, or prediction for the need of surgery (Prigent et al. 1999; Lee et al. 2018; Durand et al. 2008; Chaiwatanarat et al. 1993). Depending on the medical question, several tracers are available to determine different kidney function parameters (Taylor 2014), while SF, MTT, and OE can be determined simultaneously from a single dynamic and planar scan with the radiotracer Technetium-99 m-mercaptoacetyltriglycine (MAG3).

The radioactive glucose analog 2-deoxy-2-[^18^F]fluoro-D-glucose (FDG), routinely used mainly for oncological positron emission tomography (PET), also participates in relevant renal processes. FDG is accumulated in the renal parenchyma via the *vasa afferentes*, and a significant portion is excreted into the bladder (Scafoglio et al. 2015; Landau et al. 2007). It therefore appears obvious to determine kidney function parameters from dynamic renal FDG scans related to these processes, such as SF, MTT, and OE. Dynamic PET can be acquired during the first minutes after injection (p.i.), and kidney function could potentially be derived from a routine oncological FDG PET scan, which would save time and applied radiation dose on patients. This is of interest for patients, where kidney health status needs to be examined, e.g., in the case of intended nephrotoxic.

So far, the analysis of renal FDG behavior mainly focuses on FDG excretion, because the activity deposit in the bladder first of all is not further available for tumor identification (Garbarino et al. 2013), and secondly, it might interfere with abdominal abnormalities (Moran et al. 1999). While the kidney function parameters glomerular filtration rate and effective renal plasma flow were assessed with renal FDG TACs in our previous study (BK Geist et al. 2018), to our knowledge, SF, MTT, and OE have not yet been determined with renal FDG TACs.

For this study, a fully integrated PET/MRI system was used, offering the possibility to perform simultaneous PET and magnetic resonance imaging (MRI) acquisitions in a single scan. The high-resolution MRI sequences were used to delineate different volume-of-interest (VOIs), which were then used to extract the FDG concentration over time from the dynamic PET acquisition after image fusion.

## Materials and methods

Twenty-four adult and healthy subjects (17 males, 8 females; average age 39 ± 14 years) have been recruited and examined at the General Hospital in Vienna between January and November 2016. They fulfilled the requirements for the examinations (healthy condition, no metal in body, no claustrophobia, no pregnancy, age > 18 years). Informed consent was obtained from all individual participants included in the study. All study participants underwent MAG3 renal scintigraphy to obtain the reference values SF_ref_, MTT_ref_, and OE_ref_ as well as a 30-min FDG PET/MRI scan to obtain SF_FDG_, TT_FDG_, and OE_FDG_.

### Examination protocol

Dynamic planar renal scintigraphy was performed according to the EANM standardized protocol (Gordon et al. 2011). Volunteers were hydrated with water (10 ml/kg body weight) for 20 min and asked to empty their bladder directly before injection of around 80 MBq of MAG3. Images were acquired with a gamma camera equipped with a low-energy collimator with a frame rate of 10 s/frame for 120 frames. For further details, see Geist et al. (2015).

**Fig. 1:**
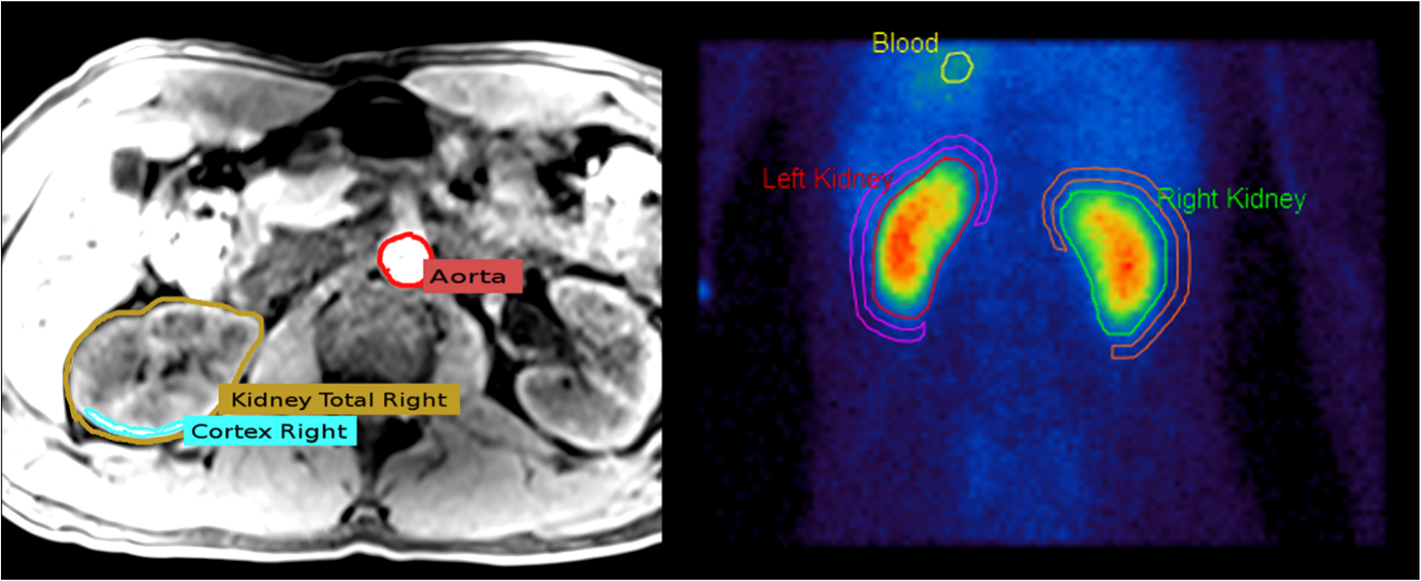
Left: Schematical illustration of the volumes of interest (VOIs) chosen in the T1 MRI sequence: total kidney region (selected in each layer), cortex region (chosen with a threshold tool in several layers), and aorta (taken from the upper part of *aorta descendens*). The aorta region in this figure does not correspond to the chosen part of the *aorta descendens*; it is only seen for illustration purposes. Right: Regions of interest (ROIs) in the planar MAG3 scintigraphy from the blood pool (heart), the kidneys, and their background

**Fig. 2:**
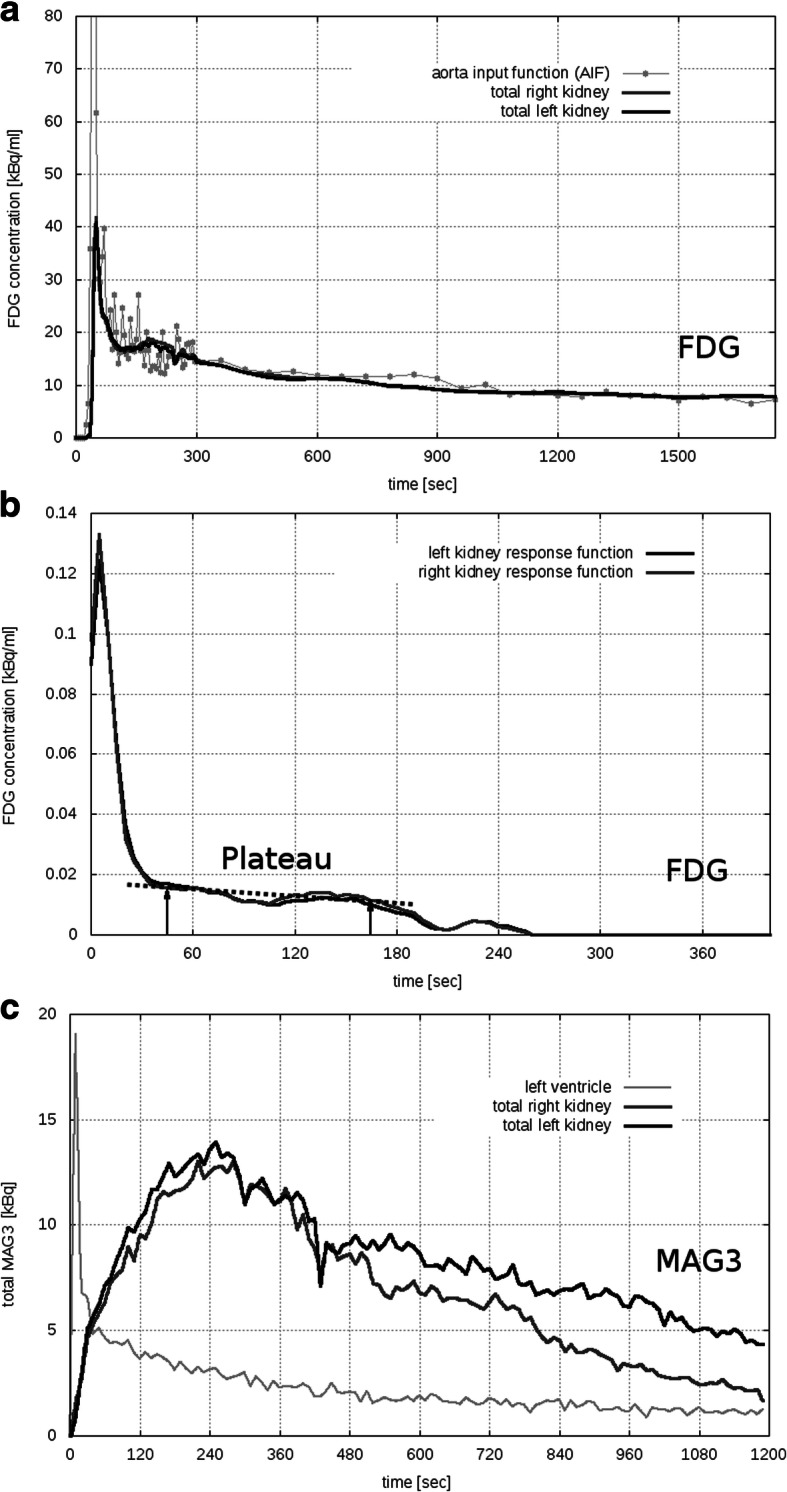
Time-activity curves. **a** Typical FDG time-activity curves (TAC) of total left and right kidney and aorta input function (AIF). For the sake of visibility, the AIF peak is cut off (maximum at 201 kBq/ml). **b** FDG response function of the total left and right kidney. **c** Background corrected MAG3 TACs of left and right kidney and the input function from the left ventricle

Extraction of MAG3 time-activity curves (TACs), see Fig. 2c, from the dynamic data, was done with *Hermes Renogram Analysis* (Hermes Medical Solutions AB, Stockholm, Sweden). Regions of interest (ROIs) were drawn around the left ventricle to obtain an input function, as well as around the kidneys and the perirenal background for background corrections of the kidney TACs (see Fig. 1).

For the PET/MRI examinations, the patient preparation was identical to the scintigraphy. With a combined PET/MRI scanner (Siemens Biograph mMR, Siemens Healthcare Diagnostics GmbH, Germany), PET acquisition started immediately after tracer injection and was continued for 30 min. The PET list-mode data was re-binned into a dynamic sequence: 60 × 5 s, 25 × 60 s, and each PET frame was reconstructed (Siemens e7 tools) into a 172 × 172 × 127 matrix using the ordinary Poisson ordered subset expectation maximization (OP-OSEM) 3D algorithm (3 iterations, 21 subsets, Gaussian filter). Scatter correction along with Dixon-based MR-attenuation correction was performed. The MRI imaging protocol consisted of a T1-weighted MRI sequence (axial breath holding and fat suppression, VIBE SPAIR). A contrast-enhanced (Dotarem 0.2 ml/kg body weight) TWIST dynamic MR sequence was performed on ten subjects, see Table 1 subject group 2. To perform quantitative analysis, five volumes of interest (VOIs) were chosen with the *Hermes Hybrid Viewer* tool (Hermes Medical Solutions AB, Stockholm, Sweden): (1) *aorta descendens* (between diaphragm and arteria renalis), drawn by hand in several layers, (2) left kidney, (3) right kidney, (4) left kidney cortex, and (5) right kidney cortex. VOIs (2–3) were carefully drawn by hand in each layer; VOIs (4–5) were delineated in about 30% of all layers by threshold ROI selection tool in the outer part of the parenchyma (see Fig. 1). VOIs were then copied to the PET images, from which the FDG TACs, i.e., the FDG concentration in the VOIs over time (Fig. 2a), were exported in units of kilo-becquerel per milliliter (kBq/ml).

**Table 1:** Subject demographics: Basic subject data presented as mean value ± standard deviation; range from minimum to maximum value in parentheses. Subject group 1 was selected for reproducibility checks, subject group 2 to estimate aorta correction effects

	All subjects *N* = 24	Reproducibility subgroup 1 (*n* = 3)	Aorta correction subgroup 2 (*n* = 10)
Gender (m/f)	18/6	1/2	6/4
Age [years]	39 ± 14 (21–65)	42 ± 18 (31–63)	42 ± 17 (21–65)
Weight [kg]	85 ± 18 (50–120)	87 ± 18 (72–107)	78 ± 11 (61–161)
Height [cm]	180 ± 9 (161–200)	178 ± 4 (175–182)	179 ± 12 (161–200)
Creatinine [mg/dl]	0.9 ± 1.16 (0.54–1.21)	1.02 ± 0.28 (0.7–1.21)	0.84 ± 0.18 (0.54–1.07)

The delineation of the ROIs in the MAG3 images was performed by a specialist for nuclear medicine (AS), and the delineation of the VOIs in the FDG images was done by a physicist (BKG).

### Determination of kidney function parameters

SF was determined by calculating the integrals from originally measured total kidney left and right TACs for minutes 1 to 2 and minutes 1 to 3, denoted as SF(1–2) and SF(1–3), respectively. Split function was expressed in percent of the left kidney. In the case of FDG, SF_FDG_ was also calculated for cortex TACs.

For MTT, the renal retention function of a corresponding TAC is needed to be determined (Durand et al. 2008). This was done with a matrix deconvolution algorithm according to Kempi (1987). To reduce noise, a Savitzky-Golay filter (Savitzky and Golay 1964) was applied on the TACs before deconvolution. An example of a left and right total kidney retention function is shown in Fig. 2b. MTT was then calculated for total left and right kidney by dividing the area under retention function by the height of the plateau (Fleming 1988; Chaiwatanarat et al. 1994). Again, for MTT_FDG_, also, cortical values were determined.

OE is defined as the total output up to time *T*, expressed as a percentage of the total input up to that time. In case of MAG3, this is usually calculated by fitting the rising part of the background corrected total kidney TAC to the integral of the input function and then multiplying the value of the TAC at time *T* with a factor obtained from the fit (Chaiwatanarat et al. 1993), which was done automatically by the *Hermes Renogram Analysis* software. Due to its sharp initial peak (Fig. 2a), the FDG TAC does not have enough rising data points to allow a valuable fitting procedure, which inhibits an application of this method. Instead, the strong linear correlation between OE_ref_ and MTT_ref_ (as reported by Fleming and Kemp (1999)) was used to convert MTT_ref_ to OE_ref_ by linear regression. The resulting slope and intercept were used to finally calculate OE_FDG_ from MTT_FDG_.

The analysis was performed by one of the authors (BKG).

### Errors

The determination of the FDG errors was done by assessing the reproducibility of the FDG TAC analysis (BK Geist et al. 2018). This was done with patient group 1 (see Table 1), consisting out of three patients with different age, gender, and kidney parameters according to the reference values. The above-described calculation methods for SF, MTT, and OE calculation were repeated using three different VOIs for blood pool and kidney in each subject. Standard errors were then calculated from all different VOIs and finally averaged over all three subjects.

### Estimation of AIF correction effects

Ten subjects who underwent an additional TWIST dynamic MRI (subject group 2, see Table 1) were used for this analysis. After image fusion, a blurring of the FDG signal was observed around the aorta region, mainly due to motion and partial volume effects, leading to an underestimation of FDG concentration. The effect was studied on the basis of a method described by Khalighi et al. (2017a, 2017b) with the data set of the subjects from subject group 2 (see Table 1) who additionally had contrast-enhanced MRI examination. According to the procedure described in BK Geist et al. (2018), the MRI images were used to determine an aorta volume, for which the FDG concentration was corrected for each frame to obtain a corrected AIF. MTT_FDG_ and OE_FDG_ from cortex and total kidney TACs of each subject was then re-calculated with the corrected AIF. The deviation of the values obtained from the corrected AIF to the uncorrected AIF was calculated, averaged over all subjects, and expressed in percent.

No motion or partial volume correction was considered for the MAG3 reference measurements.

### Statistical evaluation

Statistical analysis was performed with Gnumeric (open source software, version 1.12.20) and LibreOffice Calculator (open source software, version 6.0.3.2). First, reference values, values from FDG TACs, and basic subject data were tested for normal distribution with the Kolmogorov-Smirnov test. Correlations have been calculated with Pearson product-moment correlation coefficient *r* from which *p* value was derived. The significance of differences between reference and FDG value was assessed by a paired Student’s *t* test (*p* < 0.05 was considered as a statistically significant difference).

## Results

Eighteen male and six female patients with an average age of 39 ± 14 were examined twice, see Table 1. The averaged time period between MAG3 scan and PET/MRI scan was 9 ± 5 days. Total evaluation time for all examinations per subject was around 30 min (evaluation of scintigraphy 10 min, defining VOIs in MRI images 15 min, exporting and automatically analyzing FDG TACs 5 min). One male subject had horseshoe kidneys, and in another male subject, Tarlov cysts were incidentally found in the lower back, both not affecting kidney function. The Kolmogorov-Smirnov test showed a possible normal distribution only for SF. A correlation of *r* = − 0.90 between OE_ref_ and MTT_ref_ was found.

### Kidney function parameters

**Fig. 3:**
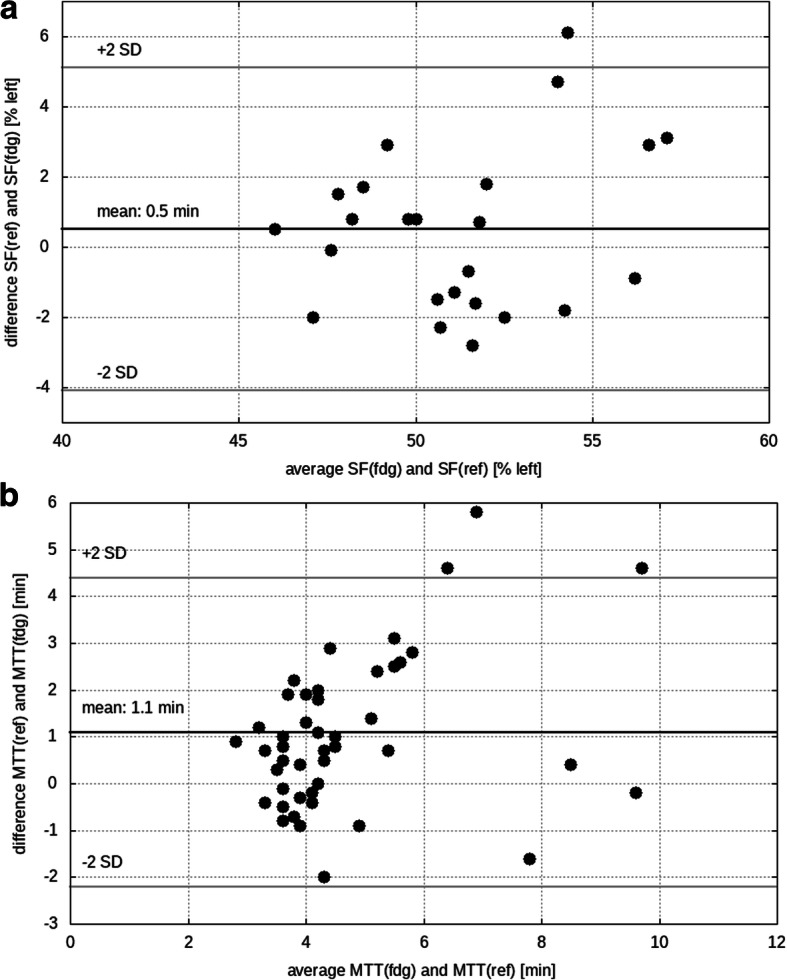
Bland-Altman analysis of (**a**) the split function (SF) for 1 to 3 min in case of the reference (ref) tracer MAG3 and for 1 to 2 min in case of FDG, and (**b**) the mean transit time for FDG and reference. Gray lines show 2 standard deviations (SD)

For total kidney, there was a good correlation between both SF_FDG_(1–2) and SF_ref_(1–2) (*r* = 0.71) and between SF_FDG_(1–3) and SF_ref_(1–3) (*r* = 0.72). Best correlation was found between SF_ref_(1–3) and SF_FDG_(1–2) with *r* = 0.75 and a mean difference of 0.5 ± 2.3% which was not significant (*p* = 0.33). The inter-correlation between SF_FDG_(1–2) and SF_FDG_(1–3), as well as between SF_ref_(1–2) and SF_ref_(1–3), was *r* = 0.99. Results for total kidney SF_FDG_(1–2) and SF_ref_(1–3) are presented in Fig. 3a.

Total kidney MTT_FDG_ correlated with MTT_ref_ (*r* = 0.74), with a significant difference between their means *p* < 0.0001 of 1.1 ± 1.6 min, see Fig. 3b.

**Fig. 4:**
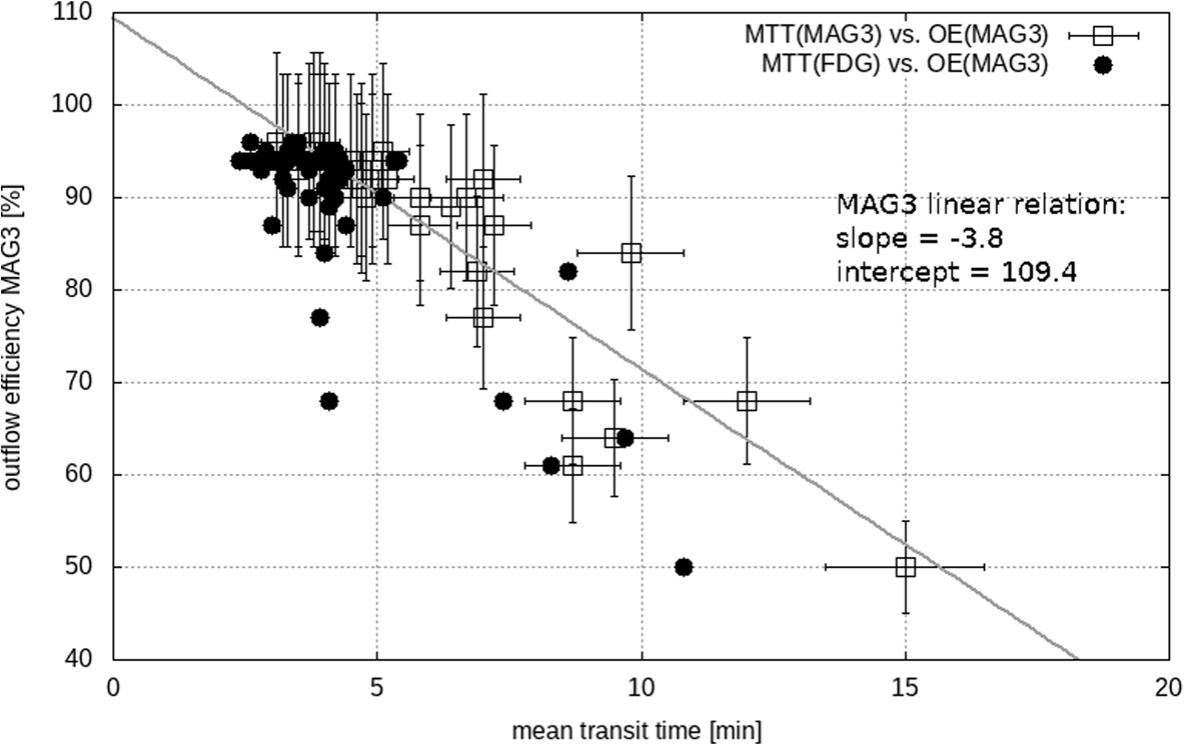
Outflow efficiency (OE) of the reference (ref) tracer MAG3 versus MAG3 mean transit time (MTT), squares, and versus FDG, circles. Correlation between MAG3 OE and MTT was − 0.90; the slope and intercept of the linear regression is also shown. Correlation between MAG3 OE and FDG MTT was − 0.81

Due to the strong linear correlation (*r* = − 0.90) between OE_ref_ and MTT_ref_, a linear regression was applied with a slope of − 3.8 and an intercept of 109.4, see Fig. 4 (rectangles). This was used to convert MTT_FDG_ to OE_FDG_, leading to a correlation of 0.81 between OE_FDG_ and OE_ref_ and a significant difference (*p* < 0.0001) between their means of − 4.1 ± 6.1% for the total kidney. Note that the correlation between MTT_FDG_ and OE_ref_ was also 0.81.

No correlations with *r* > 0.2 were found between reference values and FDG values from cortex TACs.

Reproducibility checks showed a variation of 6% for total kidney SF_FDG_ (both integrals), 9% for total kidney MTT_FDG_, and 5% for OE_FDG_, as summarized in Table 2.

**Table 2:** Results of total kidney values: mean values ± standard deviation (SD) as well as total errors, correlation coefficient (*r*), differences from Bland-Altman analysis (BA), and the *p* values from the Student’s *t* test. Displayed parameters are outflow efficiency (OE), mean transit time (MTT), and split function (SF); the latter for the integral 1 to 2 min in case of FDG values, and 1 to 3 min in the case of reference values

	Mean ± SD (min-max)	Error (%)	*r*	BA difference	*p* from *t* test
SF reference value (% left)	52 ± 4 (46–59)	10	0.75	0.5 ± 2.3	0.33
SF FDG	51 ± 3 (46–57)	6			
MTT reference (min)	5.4 ± 2.4 (3.1–15.0)	10	0.74	1.1 ± 1.6	< 0.0001
MTT FDG	4.3 ± 1.8 (2.4–10.8)	9			
OE reference (%)	89 ± 10 (50–96)	5	0.81	− 4 ± 6	< 0.0001
OE FDG	93 ± 7 (68–100)	5			

### Estimation of aorta correction effects

The effect of the observed blurring of the FDG distribution in the aorta region was estimated with a corrected AIF in subject group 2. In total, MTT_FDG_ varied overall with 4% and OE_FDG_ with − 0.2%. Note that SF_FDG_ was not affected by this correction because its calculation did not depend on the AIF.

## Discussion

The main result of our study is that SF, MTT, and OE can be estimated from renal and aortic FDG TACs obtained by dynamic PET/MRI scans.

FDG excretion from TACs of kidneys, aorta, and bladder was previously studied (Garbarino et al. 2013; Garbarino et al. 2014) on the basis of complex kinetic models. Using optimization algorithms, an excellent correlation (*r* = 0.95) has been found between the rate coefficient associated with GFR and urinary clearance. No further kidney function parameter was subtracted out of the data. In another study, the FDG clearance in the described method to correct partial volume effects (Fleming and Kemp 1999) mice was calculated by the ratio of the total renal excreted FDG activity in the bladder and the integral of the left ventricle TAC (Schnoeckel et al. 2008). Although the FDG clearance correlated well with the MAG3 tubular extraction rate (*r* = 0.73), this model was not applicable in our study due to the required extensive field of view. Furthermore, a method applied on mice under anesthesia cannot be directly transferred to humans. In a human study by Qiao et al. (2007), FDG excretion was calculated using a simple unidirectional two-compartment model. The corresponding rate constants varied significantly between the examined seven subjects and were not compared to any reference values.

Our aim was to prove that the routinely determined kidney function parameters SF, MTT, and OE can be estimated without complex models or fitting algorithms, but with standard techniques as used for MAG3 scintigraphy. Although there are pharmacological differences between MAG3 (Itoh 2001) and FDG (Scafoglio et al. 2015; Landau et al. 2007), all three parameters showed a good correlation of at least 0.74 between total kidney FDG and MAG3 reference values.

In the case of SF, no significant difference between FDG and MAG3 was found. A correlation of 0.75 was found, with SF_ref_ being within a very narrow range of 46 to 57%. Furthermore, as it was shown previously, background subtraction is a crucial issue for MAG3 planar scintigraphy, in particular for the determination of SF (Geist et al. 2015; Wesolowski et al. 2016). The uncertainty which is introduced by the background subtraction became also noticeable when the errors of the FDG and the reference values are compared, being much lower in case of FDG.

MTT_FDG_ was in average 1.1 min lower than in case of MAG3, a significant difference which can be explained with the pharmacological differences of the tracers. MTT_FDG_ showed the highest error, most probably also because the deconvolution algorithm needed for the calculation is very sensitive to small variations in the algorithm process or TAC shape (Rajabi et al. 2003).

OE_FDG_ was calculated in a different way compared to OE_ref_ as calculated by the standard method based on a TAC fit (Chaiwatanarat et al. 1993), because the FDG TAC due to its sharp peak has not enough rising data points to allow a fitting procedure. The linear relation between OE_ref_ and MTT_ref_ was used to calculate also OE_FDG_ from MTT_FDG_. Consistent with the difference in case of MTT, FDG shows a significantly higher outflow efficiency of 4%, most probably related to the different renal behavior. Certainly, the renal processes of MAG3 and FDG are not identical, but they still both undergo a renal transit as well as a renal excretion. Nevertheless, MTT_FDG_ apparently is also related to an excretion process since OE_ref_ and MTT_FDG_ correlate with 0.81.

While the evaluation of a planar scintigraphy suffers from a reliable background subtraction, this issue is indeed of little relevance in 3D FDG-PET images, but the PET scan in turn was time-consuming because a VOI must be defined out of ROIs drawn in every single layer by hand. However, the reproducibility checks have shown that even inaccurately drawn VOIs lead to maximum errors of 10%; therefore, less time-consuming assisting tools such as interpolation of ROIs can be used for VOI definition. Furthermore, blurring effects of the FDG distribution around the aorta showed a negligible effect; a sophisticated AIF correction might be disregarded for the calculation of FDG kidney function parameters.

In summary, while SF is comparable for FDG and MAG3, OE and MTT are not identical. However, the differences are minimal and the correlations are—even for the narrow value range—good; therefore, FDG might be used to at least assess the health condition of the kidneys.

### Limitations

The current study was based on healthy subjects; since the method is based on a comparison with a tracer having different properties, the efficiency of the method needs to be proven in patients with kidney disease. Furthermore, MAG3 scintigraphy was performed with 10 s/image and in a planar way (i.e., the background has to be taken into account), while for the FDG PET scans, smaller time window had to be chosen for reconstruction in order to reproduce the complex TAC shape more accurately (5 s/image during the first 5 min). Since this issue is only relevant for the deconvolution algorithm for MTT calculation and both, MTT_ref_ and MTT_FDG_ show a good accordance; this issue might be negligible.

The described method to correct partial volume effects (Fleming and Kemp 1999) in the aorta might only be applicable to PET/MRI scans, for which this method was proven to be reliable with even thinner cervical arteries used for the AIF.

Also, the variations in OE_FDG_ and MTT_FDG_ from the reference values hamper an exact determination of the evolution of the kidney health state especially in crucial cases or in follow-up studies.

Regarding a possible transfer of our method to different PET modalities, we focused in the present study on an evaluation of PET data in comparison to reference values. Due to this reason, the MRI part of our PET/MRI system was only used to separate renal cortex from other renal parts, even if several methods exist to obtain reliable values for kidney function parameters such as MTT, renal plasma flow, glomerular filtration rate, or SF (Dujardin et al. 2005; Bane et al. 2016; Claudon et al. 2014) with different sequences and with contrast agents.

## Conclusion

In case of healthy subjects, a proof of principle study was performed. The kidney function parameter split function can be determined accurately during a single dynamic FDG PET/MRI routine scan. Mean transit time and outflow efficiency show small deviations from the reference methods but might be at least estimated with FDG.

## Abbreviations

AIF: Aorta input function
BA: Bland-Altman
FDG: 2-Deoxy-2-[18F]fluoro-d-glucose
MAG3: 9mTc-labeled mercaptoacetyltriglycine
MTT: Mean transit time
OE: Outflow efficiency
PET/MRI: Positron emission tomography/magnetic resonance imaging
SF: Renal split function
TAC: Time-activity curve
